# Stepping up to the moment: collaborating on a data management and sharing workshop series

**DOI:** 10.5195/jmla.2025.2070

**Published:** 2025-07-01

**Authors:** Sara M. Samuel, Yulia V. Sevryugina, Mark MacEachern, Kate Saylor, Rachel Woodbrook

**Affiliations:** 1 henrysm@umich.edu, Taubman Health Sciences Library, University of Michigan, Ann Arbor, MI; 2 yulias@umich.edu, University Library, University of Michigan, Ann Arbor, MI; 3 markmac@umich.edu, Taubman Health Sciences Library, University of Michigan, Ann Arbor, MI; 4 kmacdoug@umich.edu, Taubman Health Sciences Library, University of Michigan, Ann Arbor, MI; 5 woodbr@umich.edu, University Library, University of Michigan, Ann Arbor, MI

**Keywords:** Data education, Data Management, Data management and sharing plans, data sharing, data services, library workshops, Workshops

## Abstract

**Background:**

Many researchers benefit from training and assistance with their data management practices. The release of the Office of Science and Technology Policy's Nelson Memo and the National Institutes of Health's new Data Management and Sharing Policy created opportunities for librarians to engage with researchers regarding their data workflows. Within this environment, we—an interdisciplinary team of librarians and informationists at the University of Michigan (U-M)—recognized an opportunity to develop a series of data workshops that we then taught during the summer of 2023.

**Case Presentation:**

The series was primarily aimed at graduate students and early career researchers, with a focus on the disciplines served by the authors in the Health Sciences - Science, Technology, Engineering, and Mathematics (HS-STEM) unit of the U-M Library. We identified three topics to focus on: data management plans, organizing and managing data, and sharing data. Workshops on these topics were offered in June, July, and August 2023.

**Conclusion:**

The number of registrants and attendees exceeded our expectations with 497 registrations across the three workshops (174/169/154, respectively), and 178 attendees (79/49/50, respectively). Registrants included faculty, staff, students, and more, and were primarily from the health sciences clinical and academic units. We received a total of 45 evaluations from the three workshops which were very positive. The slides and evaluation forms from each workshop are available through U-M's institutional repository. We developed these workshops at an opportune time on campus and successfully reached many researchers.

## BACKGROUND

Scientific and biomedical researchers need opportunities to improve their understanding of and skills in data management and sharing. In a recent survey of scientists, Tenopir et al. found a “need for organizations to offer more formal training and assistance in data management to scientists, or to better publicize the support they do offer” [1]. In another survey of biomedical researchers, it was found that most had no formal training on the topic of writing data management plans [2]. Moreover, interviews with National Institutes of Health (NIH) intramural researchers conducted by Syn and Kim found that “it is clear that institutions will benefit from increased awareness and training, ensuring researchers know their options for managing their data with an eye towards long term data preservation” [3].

Librarians and information professionals have provided and assessed instruction on research data management and sharing topics at many institutions. Xu et al. found 124 articles published between 2011 and 2021 that discuss research data management instruction in academic libraries [4]. In a case report detailing how they conducted a long-term evaluation of workshops, LaPolla, Contaxis, and Serkis reported offering workshops on data topics including data management at their institution [5]. In another case study, Read provides an overview of developing research data management education for those working in a clinical setting [6]. Rod, Hervieux, and Lee investigated an active learning intervention within the setting of introductory research data management workshops at their institution [7]. Our case study builds on this literature by illustrating how it can be important to harness current events (both on campus and in the broader research landscape) when conducting outreach or developing new educational offerings.

The University of Michigan (U-M) is one of the largest research institutions in the United States, with total research expenditures of $1.86 billion USD in fiscal year 2023, with more than $1 billion USD (56%) of this from federal funders [8]. Correspondingly, U-M has an extensive population of researchers who create, manage, and share data as a necessary part of their work. Research data services have been available at the U-M Library in some form for over a decade, including workshops to help researchers better plan for, access, manage, and share data. However, the summer of 2023 was a uniquely advantageous time at U-M for librarians and informationists to do outreach on campus about data management and data sharing as it was preceded by the introduction of the new Research Data Stewardship Initiative (RDSI) on campus, the release of a data-related memo from the Office of Science and Technology Policy (OSTP), and the new NIH Data Management and Sharing Policy going into effect.

RDSI is a campus-wide initiative focused on research data management and sharing. The initiative's goal is “[t]o ensure researchers across the University of Michigan are better positioned to equitably and securely maximize the impact of their research data” [9]. The initiative is led by the Office of the Vice President for Research and consists of a working group that includes representatives from various campus units that are involved in data management or sharing in some way, including information technology, the sponsored projects office, the research integrity office, the U-M Medical School's data office, the U-M Medical School's regulatory affairs office, and the library. RDSI was formally announced to the campus via email and newsletter in June 2022 [10], and an informational webinar was held in September 2022. RDSI then engaged the campus with a series of online seminars in which faculty from different departments presented about their involvement with—or research on—good data management and sharing practices. In June 2023, concurrently with our workshop series, RDSI announced a new institutional Research Data Stewardship Policy, which includes guidance for sharing and long-term preservation of research data [11].

Likewise, initiatives from federal scientific agencies further highlighted the importance of research data management. The Nelson Memo from OSTP, released in August 2022, sought to “make publications and their supporting data resulting from federally funded research publicly accessible,” accomplished by directing federal agencies to update their policies of how they will achieve this and resulting in researchers needing to plan for and share more of their data [12]. Both RDSI and the library harnessed the release of the Nelson Memo to increase campus awareness about growing data sharing requirements from the federal government. Moreover, the NIH's new Data Management and Sharing Policy went into effect in January 2023 [13]. In preparation, there were efforts on campus during the fall of 2022 to communicate the new requirements including information sessions, faculty meeting presentations, and the creation of online resources and videos [14]. RDSI and the library led much of this outreach.

Given the increasing campus discussions about data management and sharing resulting from RDSI, the Nelson Memo, and the new NIH policy, there was a heightened level of interest in data management and sharing topics from researchers across campus. Within this environment, we—an interdisciplinary team of librarians and informationists—recognized an opportunity to merge our expertise and develop a series of data workshops which we taught during the summer of 2023 to a receptive audience.

## CASE PRESENTATION

**Figure 1:**
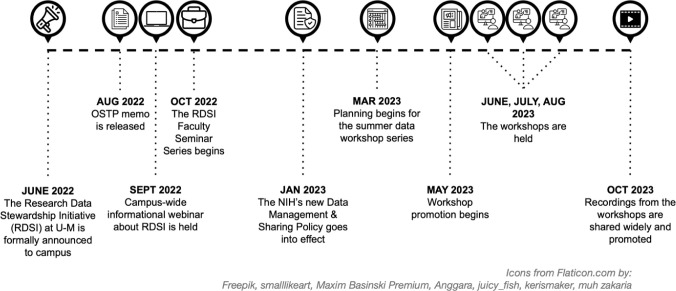
Timeline of context and key points in our work to develop and teach a summer data workshop series.

Our team consisted of a chemistry librarian, three health sciences informationists, and a data curation specialist from the institutional repositories and research data services unit in the library. The team formed in March 2023 to begin planning for the workshop series. See Figure 1 for a timeline of the context and key points in our work.

The series was primarily aimed at graduate students and early career researchers, with a focus on the disciplines served by the authors in the Health Sciences - Science, Technology, Engineering, and Mathematics (HS-STEM) unit of the U-M Library. We identified three topics to focus on: writing a data management plan, organizing and managing data, and sharing data. Workshops on these topics were scheduled in June, July, and August 2023. Each team member volunteered to plan and co-teach at least one workshop that aligned with their interests and expertise, with two instructors leading each workshop.

We began promoting the workshop series in May, sharing widely across campus in a variety of formats and utilizing existing department- and school-level communication channels. Liaison librarian colleagues were informed about the series so they could share it with their departments. We proactively reached out to relevant communities through email lists and created a graphic for digital displays in the library and other university spaces. We submitted write-ups about the series to various channels including the Medical School's newsletter submission form, which reaches thousands of researchers through different departmental newsletters. A few colleagues shared about the workshop on social media channels. See Appendix A for the graphic created to help advertise the workshops. We also referenced the entire series in each individual workshop description, as well as at the beginning and end of each workshop. We wanted it to be clear that the workshops were connected to one another and encourage attendees to register for future sessions in the series, when applicable.

The order of the workshops was a deliberate choice, since we hypothesized that many researchers would be most attracted to the workshop about writing a data management plan if they had a new need to write one due to a funder requirement or had been hearing about the new NIH policy. Then, during the first workshop we could spark their interest in the additional workshop topics and encourage them to register for those. We also developed witty yet descriptive workshop names to both grab attention and convey exactly what the workshop was about.

The workshops were taught live online via Zoom and recorded. The sessions were primarily lecture-based, but participants were engaged throughout each workshop using polls, prompts to share comments in the chat, and time for questions at the end. Clear learning objectives were identified for each workshop, and we aimed to share practical steps so that attendees could immediately begin using what they learned. An evaluation survey based on the learning objectives was shared at the end of each workshop. Table 1 provides the workshop titles and learning outcomes, with more robust descriptions of each workshop available in Appendix B.

**Table 1:** List of workshop titles, dates offered, and learning outcomes.

Workshop (Date)	Learning Outcomes
*Be prepared! Writing a data management or data sharing plan * *(June 14, 2023)*	Understand the purpose and importance of a data management plan (DMP) for research projects. Learn to create a comprehensive DMP by incorporating its typical elements effectively. Know the data management and sharing policies and requirements set by the National Science Foundation (NSF) and the NIH. Apply best practices and utilize available resources to effectively draft a DMP.
*Data, data everywhere! Managing & organizing data * *(July 13, 2023)*	Recognize the importance of data organization in facilitating high-quality research. Learn recommended practices to ensure data remains well-organized and easily retrievable throughout the research process. Understand how to effectively describe data for clarity and usability. Implement practical strategies to start organizing data more effectively.
*Help! I have to share my data: Preparation for sharing and choosing a repository for long-term data storage * *(August 10, 2023)*	Understand requirements and reasons for sharing your data. Recognize what is needed for successful data sharing. Understand how to evaluate and choose a data repository. Know when to consider using U-M's repositories, and where to get local help.

## DISCUSSION

Previous data-related workshops offered by the library (since 2013) had an average of 24 attendees and a median of 18 attendees; these numbers were fairly steady even when looking at only online sessions, only sessions from the previous two years, or only DMP-specific workshops. In contrast, our 2023 workshops series average was more than double that of previous data sessions, at 59 attendees per session (79/49/50, respectively), meaning up to 178 individuals may have attended one of our workshops if there were no repeat attendees. Registration numbers provide another data point, as registrants either intended to attend or were interested in the session content; these numbers were even higher, at 174/169/154, respectively, for a total of 497 registrations, and an average of 166 registrations per workshop. Although we do not have comparable numbers of registrations from previous data-related workshops, this was higher than what we are accustomed to seeing. We believe this increase was due to the catalysts noted above, an increased number of researchers recognizing a need for support in data management and sharing, as well as our strategic utilization of existing communication channels. Attendance for the first workshop, which focused on crafting DMPs specifically, was highest, which was likely due to more people now having to write a data management and sharing plan for their NIH grant applications.

The registration system we used for our workshops captures some basic information about registrants, although it is self-reported and may be outdated if a person shifted status or positions at the university. Interest in research data management and sharing cuts across all statuses at the university since we had registrants who were students, staff, faculty, and more. A good portion of registrants for each workshop are staff, which could be due to so many staff members supporting researchers and faculty in their data activities. Also, the workshop about DMPs drew the most faculty attention, while the data management workshop was most appealing for students. See Appendix C to see the breakdown of the university status of registrants for each workshop.

In addition to university status, the registration system also includes self-reported unit affiliations. Registrants were from across the university, although primarily from the health sciences and medical units, as expected due to the new NIH requirement, and the College of Literature, Science & Arts, where most of the science units live on campus. Appendix D shows the breakdown of unit affiliations of the workshop registrants.

An evaluation survey was shared at the end of each session and via the follow-up email with the workshop slides and recording. For each workshop, the learning objectives were the main components of the evaluation, and respondents indicated their increase in understanding: no increase, minimal, moderate, or major. The U-M Institutional Review Board reviewed the evaluation survey and determined that it is not regulated (HUM00260990).

We received a total of 45 evaluations, with 23 responses for Workshop 1, five responses for Workshop 2, and 14 responses for Workshop 3, which were very positive. Overall, 46% of the responses indicated a major increase in understanding of the learning objectives, and 87% indicated at least a moderate increase. Table 2 shows the breakdown of the learning objectives and increase in understanding for each objective, and Appendix E shows a visual display of the mean increase in understanding for each.

**Table 2:** Increase in understanding for each workshop, using evaluation questions based on the learning objectives, % responses (N responses). Total responses is 45, with 23 responses for Workshop 1, five responses for Workshop 2, and 14 responses for Workshop 3.

Workshop	How much has your understanding changed as a result of this workshop?	1 - No Increase	2 - Minimal Increase	3 - Moderate Increase	4 - Major Increase	Mean level of increase (1-4) (SD)
1	What a data management plan (DMP) is and why researchers write one	0	9% (2)	44% (10)	48% (11)	3.4 (0.7)
1	Typical elements of a DMP	0	4% (1)	35% (8)	61% (14)	3.6 (0.6)
1	DMP policies from NSF and NIH	0	13% (3)	44% (10)	44% (10)	3.3 (0.7)
1	Best practices & resources for writing a DMP	0	4% (1)	35% (8)	61% (14)	3.6 (0.6)
2	Why organizing your data is important for enabling high quality research	0	60% (3)	20% (1)	20% (1)	2.6 (0.9)
2	Recommend ed practices for keeping your data organized	0	0	60% (3)	40% (2)	3.4 (0.5)
2	Ways to describe your data	0	0	60% (3)	40% (2)	3.4 (0.5)
2	Practical strategies to start organizing your data	0	20% (1)	40% (2)	40% (2)	3.2 (0.8)
3	Why share your data	7% (1)	21% (3)	43% (6)	29% (4)	2.9 (0.9)
3	What is involved in successful data sharing	7% (1)	29% (4)	57% (8)	3.4 (0.9)	7% (1)
3	Where to deposit your data	0	21% (3)	50% (7)	29% (4)	3.1 (0.7)
3	Availability of a local data repository	7% (1)	7% (1)	43% (6)	43% (6)	3.2 (0.9)

The response number was small for the second workshop, which had about a 10% response rate compared to response rates of roughly 30% for the first and third workshops. We believe this is due to the second workshop running the full hour, resulting in more people leaving the Zoom call before we shared the evaluation link.

In addition to rating their increase in understanding for the learning objectives, the evaluation survey also provided space for attendees to share comments. A few comments indicated that the workshop was a success:

“I was brand new to data management plans, and I felt like it was a very clear, well-paced overview for someone without any experience.”

- Attendee 1 (Workshop 1)

“This was really great! Thank you for putting this workshop series together. It makes the idea of writing a DMP much less daunting.”

- Attendee 2 (Workshop 1)

“This was great; thank you.”

- Attendee 3 (Workshop 3)

We also produced a video recording of each workshop. In addition to sharing the recording and slides with the registrants via email after each workshop, we posted them on a dedicated online guide page [15] and broadly advertised their availability during the following fall semester. As of January 29, 2025, the three workshop recordings have a combined total of 128 views (66/39/23, respectively). Appendix F provides an overview of numerical outcomes for each workshop.

To make the workshop materials available in a way that was adaptable and reusable for other contexts and institutions, we deposited the unbranded workshop slides and evaluation forms into U-M's institutional repository, Deep Blue Documents. The files are licensed for reuse and available to librarians and researchers at our own and other institutions [17–19]. We publicized the reusable materials via a presentation at the Midwest Chapter of the Medical Library Association's conference [20] and at the American Chemical Society's conference [21]. As of January 2025, the materials have been downloaded 485 times in total.

### Lessons Learned

The timing of our workshops was one key to our success in reaching more researchers, so we will be continuing to monitor relevant updates to funder policies in order to provide useful and timely instruction and outreach. We will continue to share our workshops broadly with the various channels that we have access to. We now know that it's best to share an evaluation form with dedicated time in the workshop for attendees to fill it out before they leave. Additionally, in reflecting on our experience, our interdisciplinary team allowed for broader outreach about the workshops and provided us with more expertise to draw from for the development of the workshops.

### Limitations

As this is a case study, we are sharing our experience with one set of workshops at our specific institution, and there are limitations. Our experience may not be replicable at a smaller institution or one that is less affected by funding requirements from federal agencies, and also because this opportunity was time-bound and would depend on a similar new large-scale data sharing requirement or initiative in the future. As noted above, the registrant status and affiliation data from the workshop registration system may not be completely accurate. We also recognize the low response rate to the second workshop's evaluation survey.

### Future Directions

We have used the workshops' slides and recordings to start conversations with researchers about how to best write their data management plans and prepare their data for sharing, and the recordings continue to receive views. Having these materials publicly available also allows us to scale up our support of the increasing number of researchers now subject to data management planning and sharing requirements, without additional time and effort from the authors or our colleagues. We have also refined and taught versions of the workshops to additional groups on campus. Building on our success, we are now planning another collaborative workshop series to share about specific data repositories that U-M researchers can use to share and preserve their data.

We took the initiative to develop these workshops at an opportune time on campus and were successful in reaching many researchers. These workshops gave us an opportunity to demonstrate the library's expertise in data management and sharing by harnessing current events and offering timely educational opportunities, as evidenced by the increased attendance and positive feedback for these sessions. We hope our experience is useful for other librarians who wish to provide effective outreach and instruction at their institutions.

## Data Availability

The workshop materials discussed in this paper, including the evaluation surveys, are available from Deep Blue Documents: Samuel S, Sevryugina YV. Be prepared! Writing a data management or data sharing plan [Internet]. 2023. Available from: https://dx.doi.org/10.7302/8680. Samuel S, Saylor K. Data, data everywhere! Managing & organizing data [Internet]. 2023. Available from: https://dx.doi.org/10.7302/8684. Woodbrook R, MacEachern M. Help! I Have To Share My Data: Preparation For Sharing and Choosing a Repository For Long-Term Data Storage [Internet]. 2023. Available from: https://dx.doi.org/10.7302/8685.
