# Supplementary material for: Stepping up to the moment: collaborating on a data management and sharing workshop series
Source: J Med Libr Assoc. 2025 Aug 1;113(3):252–8. doi: 10.5195/jmla.2025.2070 (PMC12369970; doi:10.5195/jmla.2025.2070)
Supplement: Supplementary file 1 — Appendix A [file jmla-113-3-252-s01.pdf]

Appendix A

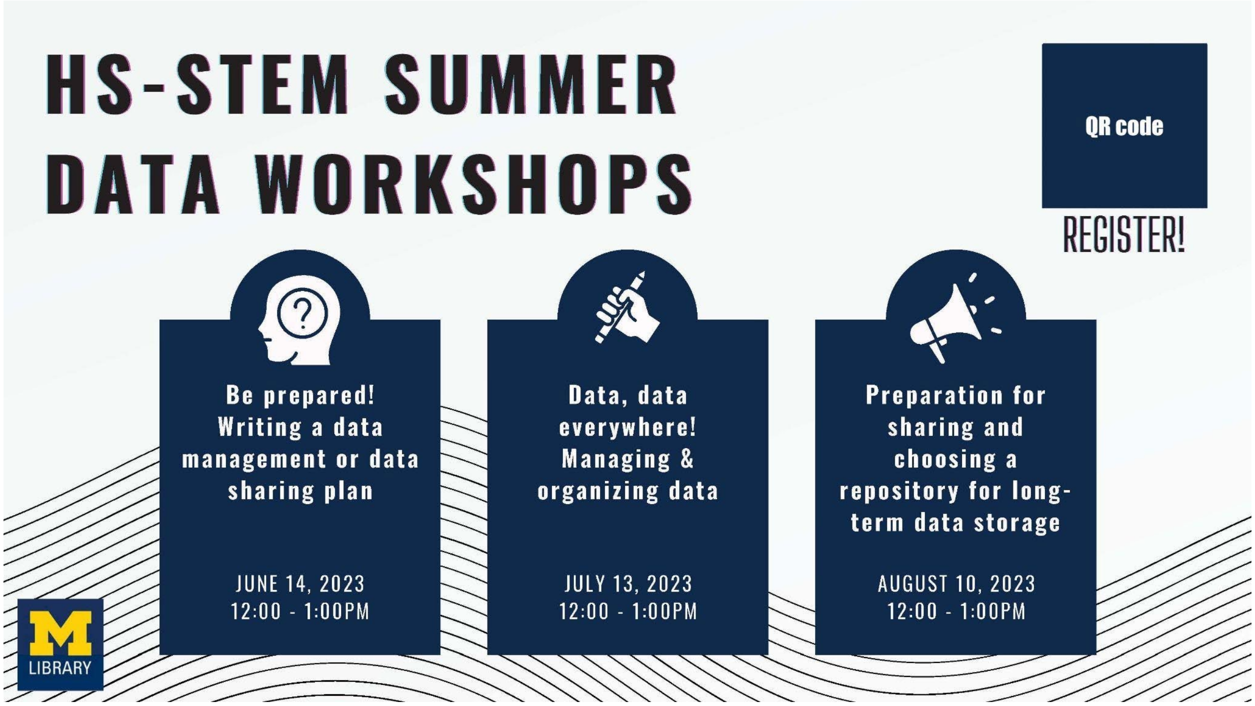

Graphic used for digital displays and social media posts to advertise the workshop series.
