# Supplementary material for: Stepping up to the moment: collaborating on a data management and sharing workshop series
Source: J Med Libr Assoc. 2025 Aug 1;113(3):252–8. doi: 10.5195/jmla.2025.2070 (PMC12369970; doi:10.5195/jmla.2025.2070)
Supplement: Supplementary file 2 — Appendix B [file jmla-113-3-252-s02.pdf]

Appendix B

Workshop 1: Be prepared! Writing a data management or data sharing plan

The objectives for the first workshop were to equip researchers with a comprehensive understanding of a Data Management Plan (DMP), and to emphasize the importance of a DMP for compliance with funding agency requirements and for enhancing data sharing and re-usability. During the workshop, we introduced attendees to each element of a DMP, showing examples that illustrated data types, metadata, storage solutions, security measures, privacy protections, reuse policies, access provisions, timelines, and oversight responsibilities. We paid particular attention to the NSF and NIH requirements for DMP in order to prepare researchers for the specific expectations from these agencies. We also highlighted common obstacles encountered when crafting a DMP, introduced the DMPTool [1], and shared best practices and resources. Participants learned what constitutes a DMP and how to develop a DMP that not only meets institutional and funder requirements, but also facilitates their own work and results in more shareable data of better quality.

Workshop 2: Data, data everywhere! Managing & organizing data

The second workshop focused on best practices in data organization, file naming, and documentation. To highlight the importance of well-organized data to research quality, we reviewed several examples of consequences for poorly managed data that contributed to significant costs and embarrassing publication retractions. We also emphasized the role of data management in upholding scientific values like transparency and reproducibility, noting a 2016 Nature survey where over 70% of researchers struggled to replicate others' experiments, often due to data management issues [2]. The workshop closed with practical strategies to assist researchers in integrating data management and documentation practices in small, manageable steps. For instance, choosing a single folder of files to implement a naming convention or outlining folder structures before starting their research. We also encouraged assigning a point person for these tasks and suggested communication practices to ensure everyone on the team understands and uses the organization system.

Workshop 3: Help! I have to share my data: Preparation for sharing and choosing a repository for long-term data storage

The third workshop focused on questions, benefits, and requirements around data sharing. These included introducing guidance and policies from NSF, NIH and OSTP, as well as new local university requirements around data stewardship and sharing [3]. We discussed additional benefits of data sharing, including increased citations and reproducibility, and the difference between data being truly available versus "available upon request." We touched on prerequisites for effective and appropriate data sharing—many of which were covered in the first two workshops—including consideration of the ethical implications of sharing or not sharing data, especially where research includes human or animal subjects, proprietary elements, or the potential for misuse. Finally, in order to move from theory to concrete resources and recommendations, we introduced criteria for defining needs and selecting an appropriate data repository, including introducing the NIH's Guidance on Selecting a Data Repository [4]. We also did a live demonstration of the U-M's institutional data repository, Deep Blue Data, discussing how to evaluate dataset eligibility and its appropriateness for public sharing, as well as outlining the support available through the U-M Library's repository and curation services, which are available to U-M researchers at no cost [5].

References

1. DMPTool [Internet]. [cited 2024 Jul 1]. Available from: https://dmptool.org/
2. Baker M. 1,500 scientists lift the lid on reproducibility. Nature. 2016 May 1;533(7604):452–4.

3. Research Data Stewardship Policy | Standard Practice Guides - University of Michigan [Internet]. [cited 2024 Jun 21]. Available from: https://spg.umich.edu/policy/303.06
4. Selecting a Data Repository | Data Sharing [Internet]. [cited 2024 Jun 21]. Available from: https://sharing.nih.gov/data-management-and-sharing-policy/sharing-scientific-data/selecting-a-data-repository#desirable-characteristics-for-all-data-repositories
5. Deep Blue Data [Internet]. [cited 2024 Jun 21]. Available from: https://deepblue.lib.umich.edu/data/
