# Supplementary material for: Stepping up to the moment: collaborating on a data management and sharing workshop series
Source: J Med Libr Assoc. 2025 Aug 1;113(3):252–8. doi: 10.5195/jmla.2025.2070 (PMC12369970; doi:10.5195/jmla.2025.2070)
Supplement: Supplementary file 3 — Appendix C [file jmla-113-3-252-s03.pdf]

Appendix C

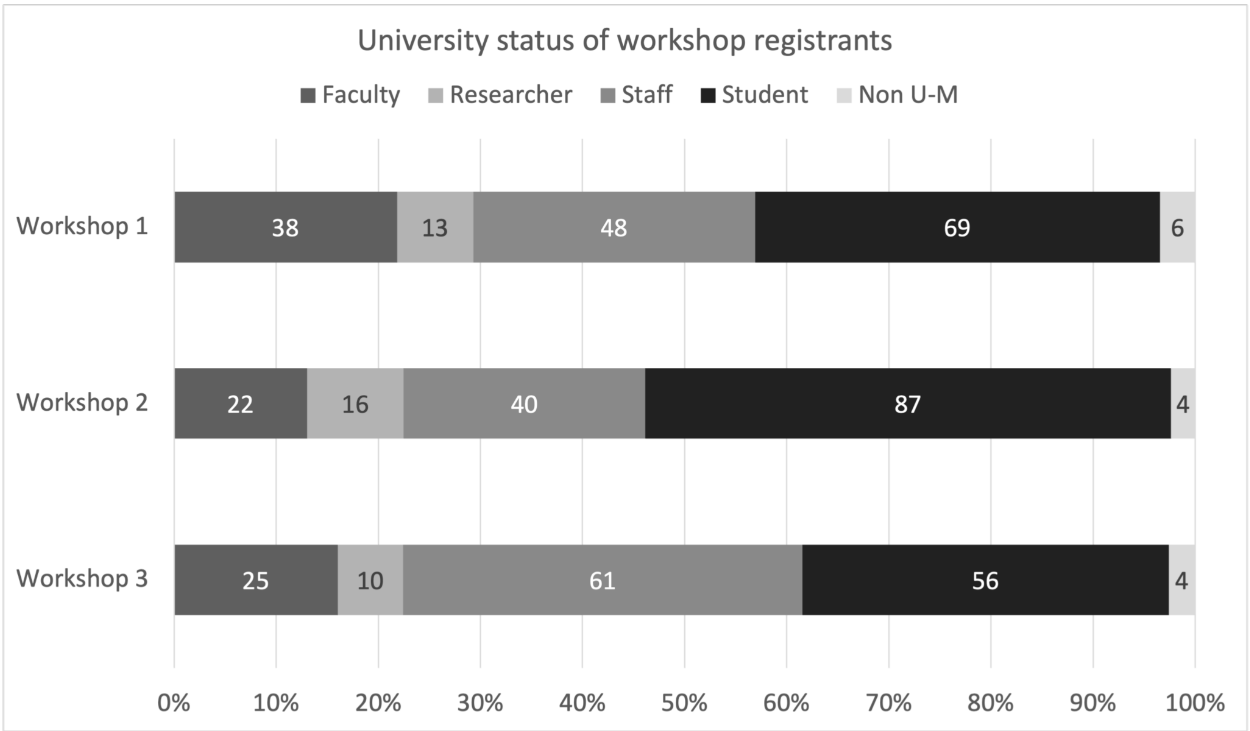

University status of workshop registrants. The total number of registrants is 497, with 174 registrants for Workshop 1, 169 registrants for Workshop 2, and 154 registrants for Workshop 3.
