# Supplementary material for: Stepping up to the moment: collaborating on a data management and sharing workshop series
Source: J Med Libr Assoc. 2025 Aug 1;113(3):252–8. doi: 10.5195/jmla.2025.2070 (PMC12369970; doi:10.5195/jmla.2025.2070)
Supplement: Supplementary file 5 — Appendix E [file jmla-113-3-252-s05.pdf]

Appendix E

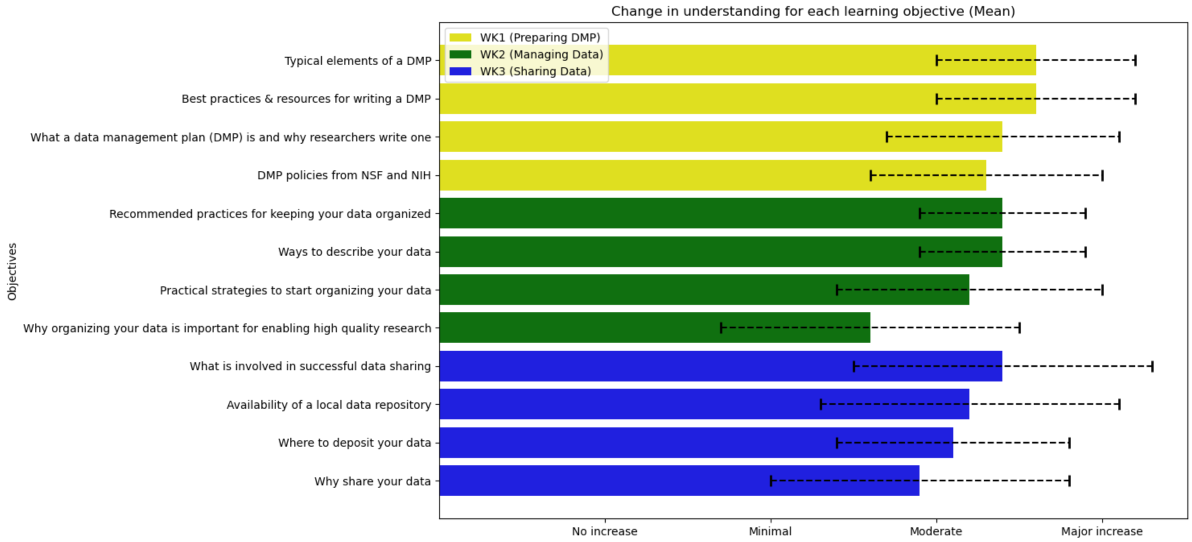

Visualization of Table 2 within the main text. Mean increase in understanding for each workshop, using evaluation questions based on the learning objectives. The total number of responses is 45, with 23 responses for Workshop 1, 5 responses for Workshop 2, and 14 responses for Workshop 3.
