# Supplementary material for: Stepping up to the moment: collaborating on a data management and sharing workshop series
Source: J Med Libr Assoc. 2025 Aug 1;113(3):252–8. doi: 10.5195/jmla.2025.2070 (PMC12369970; doi:10.5195/jmla.2025.2070)
Supplement: Supplementary file 6 — Appendix F [file jmla-113-3-252-s06.pdf]

Appendix F

Table 3. Numerical outcomes for each workshop.

	**Registrants**	**Attendees**	**Completed evaluations**	**Recording views as of January 29, 2025**
Workshop 1	174	79	23	66
Workshop 2	169	49	5	39
Workshop 3	154	50	14	23
